# Supplementary material for: Bioengineered human myobundles mimic clinical responses of skeletal muscle to drugs
Source: eLife. 2015 Jan 9;4:e04885. doi: 10.7554/eLife.04885 (PMC4337710; doi:10.7554/eLife.04885)
Supplement: Source code 1. — Source code for analysis of contractile force in MATLAB. DOI: http://dx.doi.org/10.7554/eLife.04885.023 [file elife04885s001.docx]

**analyzeForceTet20.m**

%% Analysis of tetanus amplitude at 20 Hz stimulation

clear

clc

close all

direc = uigetdir;

if direc(end) ~= '\'

direc(end+1) = '\';

end

% sampleName = 'hmsc1';

fileList = dir(direc);

fnames = {fileList.name};

fnames = fnames(3:end);

goodNames = {};

mags = {};

mins = {};

stim = {};

stimi = {};

maxi = {};

maxs = {};

check = {};

activeForce = {};

ind=1140; %THIS IS THE INDEX BEFORE THE LAST STIMULATION, ADJUST AS NECESSARY

for i = 1:length(fnames)

name = fnames{i};

if ~isempty(strfind(name,'.fmd')) && (~isempty(strfind(name,'20Hz')));

name

goodNames = [goodNames;{name}];

fh = fopen([direc name],'r');

line = fgetl(fh);

while ~strcmp(line,'abcd')

line = fgetl(fh);

end

line = fgetl(fh); % get abcd line that is at beginning of data

data = [];

while ischar(line)

data = [data str2double(line)];

line = fgetl(fh);

end

fclose(fh);

data = data(1:end/3);

minForce = mean(data(1:70));

maxStim = max(data);

lastStim = max(data(ind:1990));

lastStimIndex = ind+find(data(ind:end)==lastStim,1,'last');

%Find last stimulation peak, for 20Hz is near 1165

maxTwitch = max(data(lastStimIndex+10:end));

maxTwitchIndex = find(data==maxTwitch,1,'last');

activeForce = maxTwitch - minForce;

checkMax = maxStim-maxTwitch;

% plot(data-min(data))

check = [check;{checkMax}];

mins = [mins;{minForce}];

maxs = [maxs;{maxTwitch}];

stim = [stim;{maxStim}];

stimi = [stimi;{lastStimIndex}];

maxi = [maxi;{maxTwitchIndex}];

mags = [mags;{activeForce}];

end

end

toWrite = [{'file' 'checkmax' 'baseline force' 'max force twitch' 'max force stim' 'stim index' 'max index' 'active force (mN) twitch'};...

[goodNames check mins maxs stim stimi maxi mags]];

xlswrite([direc 'resultsTet20.xlsx'], toWrite)

**analyzeForceTwitch.m**

%% Analysis of twitch amplitude

clear

clc

close all

direc = uigetdir;

if direc(end) ~= '\'

direc(end+1) = '\';

end

% sampleName = 'hmsc1';

fileList = dir(direc);

fnames = {fileList.name};

fnames = fnames(3:end);

goodNames = {};

mags = {};

mins = {};

maxs = {};

activeForce = {};

for i = 1:length(fnames)

name = fnames{i};

if ~isempty(strfind(name,'.fmd')) && ~isempty(strfind(name,'116')) && (isempty(strfind(name,'Hz')) && isempty(strfind(name,'hz'))&& isempty(strfind(name,'FAT')));

name

goodNames = [goodNames;{name}];

fh = fopen([direc name],'r');

line = fgetl(fh);

while ~strcmp(line,'abcd')

line = fgetl(fh);

end

line = fgetl(fh); % get abcd line that is at beginning of data

data = [];

while ischar(line)

data = [data str2double(line)];

line = fgetl(fh);

end

fclose(fh);

data = data(1:end/3);

minForce = mean(data(1:90));

maxTwitch = max(data(140:500));

activeForce = maxTwitch - minForce;

% plot(data-min(data))

mins = [mins;{minForce}];

maxs = [maxs;{maxTwitch}];

mags = [mags;{activeForce}];

end

end

toWrite = [{'file' 'baseline force' 'max force twitch' 'active force (mN) twitch'};...

[goodNames mins maxs mags]];

xlswrite([direc 'resultsTwitch.xlsx'], toWrite)
